# Editorial: Advancing diagnostic excellence in early lung cancer detection

**DOI:** 10.3389/fonc.2026.1913109

**Published:** 2026-07-09

**Authors:** Roger Y. Kim, Michael N. Kammer, Yeon Wook Kim

**Affiliations:** 1 Division of Pulmonary, Allergy, and Critical Care, Department of Medicine, University of Pennsylvania, Philadelphia, PA, United States; 2 Penn Center for Cancer Care Innovation at the Abramson Cancer Center, University of Pennsylvania, Philadelphia, PA, United States; 3 Institut de Recherche en Informatique de Toulouse, Université Toulouse Capitole, Toulouse, France; 4 Division of Pulmonary and Critical Care Medicine, Department of Internal Medicine, Seoul National University Bundang Hospital, Seongnam, Republic of Korea; 5 Department of Internal Medicine, Seoul National University College of Medicine, Seoul, Republic of Korea

**Keywords:** cancer screening (MeSH), early detection of cancer, lung neoplasms, lung neoplasms - diagnosis, multiple pulmonary nodules, solitary pulmonary nodule

Lung cancer kills more people worldwide than any other cancer because of a combination of its high incidence and diagnosis at late stage (1). As such, efforts to optimize early lung cancer detection and diagnosis via 1) screening, 2) pulmonary nodule risk assessment and management, 3) lung biopsy procedural innovation, and 4) adjunctive diagnostic modalities are critically important to ultimately improve patient clinical outcomes (2). The aim of this Research Topic is to highlight recent advances in lung cancer early detection and diagnosis, with the goal of drawing much needed attention to significant clinical and scientific gaps in lung cancer diagnostic excellence (3).

The National Lung Screening Trial and Dutch-Belgian Randomized Lung Cancer Screening Trial definitively demonstrated that lung cancer screening with low-dose computed tomography among individuals with a substantial smoking history reduces lung cancer-specific mortality (4, 5). However, real-world implementation of lung cancer screening has often differed from clinical trial protocols due to a lack of health system resources, patient and clinician factors, and lung cancer stigma (6–16), despite demonstrating evidence of clinical benefit (17–19). Studies examining lung cancer screening within routine clinical care are essential to understand how best to realize the benefits of screening while identifying areas for additional improvement and research. In this Research Topic, Duncan et al. reported the impact of a screening education curriculum tailored for primary care clinicians on their knowledge of guidelines, comfort with shared decision-making, communicating results, and managing follow-up care, and Kandasamy et al. demonstrated an overall lack of knowledge and familiarity with lung cancer screening among adults living in the Asir region of Saudi Arabia while exploring factors associated with increased knowledge. Future studies are required to further explore ways to improve lung cancer screening clinical implementation.

Pulmonary nodules are frequently detected on chest computed tomography imaging either incidentally or via screening and may represent early-stage lung cancer (20). However, as most nodules are benign (21) and lung biopsy carries considerable procedural risk (22–25), cancer risk assessment is crucial for determining appropriate clinical management (26–29). Several articles included in this Research Topic sought to study pulmonary nodule cancer risk assessment. First, Chen et al. evaluated a machine learning assisted breathomic approach for early-stage cancer detection among adults with clinical suspicion of thoracic malignancy and demonstrated feasibility. Second, Huang et al. developed and tested a predictive model using clinical and imaging characteristics to estimate pulmonary nodule cancer probability. Third, Lyu et al. explored the use of ^18^F-FAPI-04 positron emission tomography for evaluation of ground-glass pulmonary nodule invasiveness. Finally, Anghel et al. authored a narrative review of pulmonary nodule monitoring strategies based on nodule features, highlighting volume doubling time as an indicator of tumor size progression.

Once the decision is made to proceed with lung biopsy for evaluation of a suspicious pulmonary nodule, various diagnostic procedures exist, including bronchoscopy, transthoracic needle biopsy, and surgical resection (2). While there have been notable recent advances in advanced navigational diagnostic bronchoscopy procedural techniques (30–32), contributions to this Research Topic focused on procedural innovations in transthoracic needle biopsy. Huang et al. assessed the impact of an active breathing coordinator combined with a custom-designed puncture needle on pulmonary nodule diagnostic accuracy and procedural complication rates. Meanwhile, Song et al. conducted a randomized clinical trial investigating computed tomography-guided coaxial needle sequence puncture with suture-anchored needle guidance and coupled needle retraction and advancement, and found increased pulmonary nodule puncture accuracy compared to conventional synchronous puncture and needle insertion.

Lastly, this Research Topic contains diverse contributions that focus on various aspects of lung cancer diagnosis, management, and clinical outcomes. Wang and Dai reviewed current progress and future directions regarding lung cancer associated with cystic airspaces, underscoring the need for a standardized approach to diagnosing and treating this unique lung cancer subtype. Zhang et al. reminded us that Rosai-Dorfman disease can clinically mimic lung cancer in their case report presentation. Vedachalam et al. demonstrated that an organizing pneumonia pattern can concomitantly present alongside lung cancer during histopathologic evaluation, suggesting that an organizing pneumonia result from minimally invasive lung biopsy should not be classified as a specific benign finding. Zhang et al. examined a T2-weighted magnetic resonance imaging grading system for preoperative prediction of lung cancer visceral pleural invasion, suggesting it may be used as a complementary imaging modality. Ye et al. assessed the diagnostic performance of probe-based confocal laser endomicroscopy in the diagnosis of malignant pleural effusion. Yang et al. reviewed the clinical applications of radionuclide theranostics in lung cancer. Ren et al. developed and validated machine learning models for predicting spread through air spaces in stage I lung adenocarcinoma presenting as part-solid and solid pulmonary nodules using both clinical and imaging characteristics. Völter et al. explored the association between pretreatment standardized value uptake on positron emission tomography and the development of immune checkpoint inhibitor-related pneumonitis in patients with lung cancer, finding no statistically significant correlation. Cai et al. developed and validated a prognostic nomogram incorporating demographic and common laboratory values to predict lung cancer overall survival. Finally, Li et al. performed a network meta-analysis to evaluate therapeutic regimens for advanced anaplastic lymphoma kinase rearrangement in non-small cell lung cancer.

All editors of this Research Topic sincerely thank all contributing authors for submitting their work and all reviewers for lending their time critically evaluating manuscripts. As an editorial team, it is our hope that this Research Topic will draw increased attention to early lung cancer detection and diagnosis and inspire others to continue working together collaboratively to improve thoracic oncology clinical care and research.
